# Chlamydialike Organisms and Atherosclerosis

**DOI:** 10.3201/eid1204.050751

**Published:** 2006-04

**Authors:** Gilbert Greub, Olivier Hartung, Toïdi Adekambi, Yves S. Alimi, Didier Raoult

**Affiliations:** * Université de la Méditerannée, Marseille, France;; † Hôpital Nord, Marseille, France

**Keywords:** Atherosclerosis, Chlamydia-like, Parachlamydiaceae, Neochlamydia hartmanellae, Parachlamydia acanthamoebae, novel chlamydiae, letter

**To the Editor:** *Chlamydophila pneumoniae* causes pneumonia, but its role in the pathogenesis of atherosclerosis is controversial (*1**–**4*). The role of *C*. *pneumoniae* in atherosclerosis is supported by seroepidemiologic studies and detection in atherosclerotic lesions by polymerase chain reaction (PCR), immunohistologic analysis, culture, and electron microscopy (*2**,**3*). However, these results were not confirmed by other serologic or PCR-based studies (*4*). Meijer et al. evaluated abdominal aortic aneurysm biopsy specimens and detected *C*. *pneumoniae* membrane antigen more frequently than lipopolysaccharide antigens but did not detect heat shock protein 60 (*1*). In addition, they could not amplify or detect specific *C*. *pneumoniae* DNA by PCR and fluorescence in situ hybridization (*1*). They hypothesized that this discrepancy may result from a chlamydialike organism present in aortic samples that has surface antigens similar to those of *C*. *pneumoniae*.

*Parachlamydia acanthamoebae* and *Neochlamydia hartmanellae* are chlamydialike organisms that share ≈86% 16S rRNA sequence similarity with *C*. *pneumoniae* (*5*). Like *C*. *pneumoniae*, they have elementary and reticulate bodies visible by electron microscopy (*6*). *Neochlamydia*-related DNA (GenBank accession no. AF097191) has been amplified from 5 different arterial samples, including 1 aortic aneurysm (*7*), and a relationship (p = 0.009) between cerebral hemorrhage and serologic evidence of *Parachlamydia* infection has been reported (*8*). Therefore, we investigated the role of *Parachlamydia* in pathogenesis of atherosclerosis by using a molecular approach.

We analyzed 78 surgical samples from 27 patients undergoing aortic or carotid surgery for atherosclerotic disease at Hôpital Nord in Marseille from June 1, 2003, to December 31, 2003. The study was approved by the local ethics committee, and written informed consent was obtained from all participants. Demographic and clinical data were prospectively recorded.

DNA was extracted from aortic or carotid samples with atherosclerotic lesions by using the QIAamp DNA tissue kit (Qiagen, Courtaboeuf, France), according to the manufacturer’s instructions. A nested PCR was performed by using external primers 16SIGF (5´-CGGCGTGGATGAGGCAT-3´) and 16SIGR (5´-TCAGTCCCAGTGTTGGC-3´) (*9*) and internal primers CHL16SFOR2 (5´-CGTGGATGAGGCATGCAAGTCGA-3´) and CHL16SREV2 (5´-CAATCTCTCAATCCGCCTAGACGTCTTAG-3´) (*7*). PCR included negative controls from the DNA extraction step. DNA extractions and PCR amplifications were conducted in a laboratory in which parachlamydial DNA had not been extracted or amplified. PCR products were purified by using the QIAquick PCR purification kit (Qiagen) and sequenced by using the d-rhodamine terminator cycle sequencing reaction kit (Perkin-Elmer Biosystems, Warrington, UK) and a 3100 ABI Prism automated sequencer (Applied Biosystems, Courtaboeuf, France).

Sequences were analyzed with BLAST (http://www.ncbi.nlm.nih.gov/BLAST/) using gap existence and extension penalties of 5 and 2, respectively. Results were considered positive only when the sequence of the amplified product exhibited a best BLAST hit with a chlamydialike organism. Statistical analyses were performed with STATA software (Stata Corporation, College Station, TX, USA).

A positive PCR result was obtained with samples from 5 (18.5%) of 27 patients (Table). Three sequences had a best BLAST hit with the sequence of *Parachlamydia* sp. UV7 (GenBank accession no. AJ715410), with a sequence similarity ranging from 99% to 100%. The other 2 sequences had 98% sequence similarity with *Neochlamydia*-related symbiont TUME-1 (GenBank accession no. AF098330). PCR positivity was not associated with age, sex, or location of the atherosclerotic lesion.

**Table:** Comparison of patients with and without parachlamydial DNA by polymerase chain reaction (PCR) in surgical aortic or carotid specimens

Characteristic	Positive PCR result (n = 5)	Negative PCR result (n = 22)	p value
Males, no. (%)	4 (80)	16 (73)	0.74
Median age (interquartile range)	72 (71–77)	66 (61–74)	0.11
Any cardiovascular risk factor, no. (%)	3 (60)	21 (95)	0.02
Hypertension	3 (60)	10 (45)	0.56
Hypercholesterolemia	0	5 (23)	0.24
Tobacco use	3 (60)	17 (77)	0.42
Diabetes	1 (20)	6 (27)	0.74
Aortic surgery, no. (%)	2 (40)	13 (59)	0.44
Previous cardiovascular disease, no. (%)	2 (40)	8 (38)	0.94

All patients with positive PCR results were >68 years of age. Patients without cardiovascular risk factors were more likely than those with >1 risk factor to have positive PCR results (p = 0.023). Despite the small number of patients in this study, this association was also confirmed in a multivariate logistic regression model adjusted for sex and previous cardiovascular disease (odds ratio 0.035, 95% confidence interval 0.001–0.94).

These findings suggest that *Parachlamydia* and *Neochlamydia* are associated with atherosclerosis. In addition, these obligate intracellular bacteria may be present in both carotid and aortic atherosclerotic lesions of elderly patients.

Chlamydialike organisms in atherosclerotic lesions may explain controversies about the role of *C*. *pneumoniae* in pathogenesis of atherosclerosis (*4*). Some PCRs might amplify both *Chlamydia* and chlamydialike organisms, leading to erroneous conclusions, especially when the specificity of the product is not confirmed by sequencing. Chlamydialike organisms in atherosclerotic lesions might also explain discrepancies of serologic studies (*4*). *Parachlamydiaceae* likely cross-react with *C*. *pneumoniae* (*1*). Such cross-reactivity will not be recognized if patients with positive serologic results for *C*. *pneumoniae* are not tested for antibodies to chlamydialike organisms. This cross-reactivity may result in false-positive serologic results for *C*. *pneumoniae*, especially when low antibody titers (8–16) are considered positive.

Since elementary and reticulate bodies are similar in both *Chlamydiaceae* and *Parachlamydiaceae* (*6*), Chlamydiales cannot be identified in a specimen solely by electron microscopy. Consequently, elementary and reticulate bodies in atherosclerotic lesions (*10*) might be chlamydialike organisms and not *C*. *pneumoniae*. If chlamydialike organisms are involved in the pathogenesis of atherosclerosis, this finding would have public health implications, given their presence in free-living amebae that are widespread in water.
